# Changes in S100A8/A9 and S100A12 and Their Comparison with Other Analytes in the Saliva of Pigs with Diarrhea Due to *E. coli*

**DOI:** 10.3390/ani13162556

**Published:** 2023-08-08

**Authors:** Alba Ortín-Bustillo, María Botía, María José López-Martínez, Silvia Martínez-Subiela, José Joaquín Cerón, Antonio González-Bulnes, Edgar García Manzanilla, Elena Goyena, Fernando Tecles, Alberto Muñoz-Prieto

**Affiliations:** 1 Interdisciplinary Laboratory of Clinical Analysis of the University of Murcia (INTERLAB-UMU), Department of Animal Medicine and Surgery, Veterinary School, Regional Campus of International Excellence Mare Nostrum, University of Murcia, Espinardo, 30100 Murcia, Spain; alba.ortinb@um.es (A.O.-B.); maria.botiag@um.es (M.B.); mariajose.lopez28@um.es (M.J.L.-M.); silviams@um.es (S.M.-S.); jjceron@um.es (J.J.C.); ftecles@um.es (F.T.); 2 Departamento de Producción y Sanidad Animal, Facultad de Veterinaria, Universidad Cardenal Herrera-CEU, CEU Universities, C/Tirant lo Blanc, 7, Alfara del Patriarca, 46115 Valencia, Spain; antoniogbulnes@cuartesa.com; 3 Cuarte S.L. Grupo Jorge, Ctra. De Logroño, Km 9,2., Monzalbarba, 50120 Zaragoza, Spain; 4 Pig Development Department, The Irish Food and Agriculture Authority, Teagasc, Moorepark, P61 C996 Fermoy, Ireland; egmanzanilla@gmail.com; 5 School of Veterinary Medicine, University College Dublin, Belfield, D04 W6F6 Dublin, Ireland; 6 Department of Animal Health, Faculty of Veterinary Medicine, University of Murcia, 30100 Murcia, Spain; goyena@um.es

**Keywords:** pigs, diarrhea, calgranulins, S100A8/A9, S100A12, saliva, *E. coli*

## Abstract

**Simple Summary:**

S100A8/A9 (also known as calprotectin) and S100A12 (also known as calgranulin C) are considered biomarkers of potential interest and are proteins members of the calgranulin family which is related to different inflammatory conditions, immune-mediated diseases, and sepsis. The objectives of this study were to evaluate if S100A8/A9 and A12 could change in the saliva of pigs with diarrhea due to *E. coli* and compare the changes of S100A8/A9 and A12 with other analytes in order to explore the possible causes or mechanisms involved in these changes. For this purpose, a panel integrated by other analytes related to inflammation, immune system, stress, tissue damage, sepsis, and redox status was also evaluated. S100A8/A9 and S100A12 increased in pigs with diarrhea produced by *E. coli* and were correlated with other salivary analytes. Further studies should be performed to elucidate the possible practical applications of these calgranulins as biomarkers to evaluate the health and welfare of pigs.

**Abstract:**

The family of calgranulins includes S100A8 (calgranulin A), S100A9 (calgranulin B), which can appear as a heterodimer known as S100A8/A9 or calprotectin, and S100A12 (calgranulin C). These proteins are related to different inflammatory conditions, immune-mediated diseases, and sepsis and are considered biomarkers of potential interest. This study aims to evaluate if S100A8/A9 and A12 could change in pigs with diarrhea due to *E. coli* and to compare the changes of S100A8/A9 and A12 with other analytes in order to explore the possible causes or mechanisms involved. For this purpose, a panel integrated by analytes related to inflammation (haptoglobin, inter-alpha trypsin inhibitor 4 (ITIH4), and total protein); immune system (adenosine deaminase, ADA); stress (alpha-amylase); tissue damage (lactate and lactate dehydrogenase (LDH)); sepsis (aldolase) and redox status (ferric-reducing ability of saliva (FRAS) and advanced oxidation protein products (AOPP)) was evaluated. S100A8/A9 and A12 and the other analytes measured in this study showed increases in the saliva of pigs with diarrhea due to *E. coli*. S100A8/A9 and/or A12 showed a significant correlation of different magnitude with some of the other analytes evaluated. Further studies should be conducted to gain knowledge about the possible practical applications as biomarkers of the measurements of S100A8/A9 and A12 in the saliva of pigs.

## 1. Introduction

The S100 proteins are a family of proteins that bind calcium, have a similar molecular mass and similarities in their amino acid sequence, and are gaining attention as biomarkers of different diseases related to the immune system and inflammation [1]. They are released to the extracellular space by damaged and/or activated cells such as neutrophils and macrophages after any cell stress or damage. Once released, they can activate immune cells and aid cytokine synthesis and inflammation events. The cell activation is made by the binding of S100 to cell pattern recognition receptors (PRRs), namely toll-like receptors (TLRs) and advanced glycation end products (RAGE) [2].

The calgranulins, which includes S100A8 (calgranulin A) and S100A9 (calgranulin B) that together form a heterodimer known as S100A8/A9 or calprotectin, and S100A12 (calgranulin C), are especially related with different inflammatory conditions, immune-mediated diseases, and sepsis [3,4,5] and are considered as biomarkers of interest in humans [6]. In many studies performed in humans, a profile including S100A8/A9 and S100A12 is studied. Although in some reports [7,8] there was a high correlation between the concentrations of S100A8/A9 and S100A12, there are some differences in these proteins. One is the fact that they can be released by different cells, therefore while SA100A8 and A9 are produced by granulocytes, monocytes, and macrophages, SA100A12 seems to be predominantly found in granulocytes [9,10]. In addition, they have different binding sites to the immune cells [11] and they can be involved in diverse physiological functions and mechanisms [7]. These differences could lead to different behavior and dynamics in these proteins and, therefore, could provide complementary information as biomarkers.

The use of saliva as a biological sample in veterinary science has become increasingly popular due to its non-invasive and easy-to-collect nature. This is particularly advantageous in the case of pigs, where taking blood samples can be difficult, stressful, and painful for the animals [12]. Saliva can be collected easily by farm staff, allowing for more frequent analysis and monitoring and subsequently better control of the health and welfare of the animals. In research, the saliva of pigs has shown utility in the detection of infectious pathogens being currently used in routine practice for this purpose [12], but it can also be used to measure biomarkers that provide insight into stress, inflammation, immune response, and redox homeostasis [12,13,14,15,16]. Therefore, saliva is a rich source of analytes that have the potential to be biomarkers of different physiopathological conditions and diseases, assess the effects of various husbandry conditions and, in general, evaluate the homeostasis of swine and provide information about its health and welfare. However, the use of saliva also has challenges such as the need, in some cases, for high-sensitivity assays for the detection of some analytes that are in low concentration in this fluid. Recently, it has been described that calprotectin can be measured in the saliva of pigs showing increases in experimentally induced sepsis [14], as well as the increases in calprotectin and S100A12 in saliva in pigs with meningitis due to *Streptococcus suis* [17]. In addition, S100A12 has been measured in human saliva in inflammatory bowel disease [18].

Enterotoxigenic *Escherichia coli* (ETEC) is one of the most common foodborne pathogens that affect piglets and weaning pigs in swine production, producing diarrhea which often results in high-rate mortality. The incidence of ETEC in the swine industry severely impacts the well-being of pigs and the economic sustainability of the sector [19]. A previous proteomic study on saliva investigated the variations of salivary proteins in pigs suffering from diarrhea due to *E. coli* [20]. In this study, several proteins were identified that varied in diseased pigs compared with healthy pigs, among them there were proteins related to the immune system like adenosine deaminase (ADA). Thus, it would be interesting to explore if calgranulins, also related to the immune response, could be changed in the saliva of pigs with diarrhea due to *E. coli*.

Despite the interest that calgranulins could have as possible biomarkers in saliva in pigs, there is still a gap in the knowledge about their behavior in different diseases and clinical conditions. In addition, there is a lack of data about their possible correlations with other different biomarkers related to inflammation, immune system, stress or redox hemostasis that could help to elucidate the physiopathological mechanisms in which calgranulins can be involved. This report hypothesizes that there could be changes in the concentrations of S100A8/A9 and S100A12 in the saliva of pigs that have diarrhea due to *E. coli* and that these changes could be potentially related to changes in other biomarkers in saliva. Therefore, the objectives of this study are (1) to evaluate if S100A8/A9 and A12 could change in a clinical condition consisting of pigs with diarrhea due to *E. coli* and (2) to compare the changes of S100A8/A9 and A12 with other analytes in order to explore the possible causes or mechanisms involved in these changes. For this purpose, a panel integrated by S100A8/A9 and A12 along with other analytes related to inflammation such as haptoglobin, Inter-alpha-trypsin inhibitor heavy chain 4 (ITIH4, also known as Pig major acute phase protein or Pig-MAP), and total proteins, analytes related to the immune system such as ADA, to stress such as alpha-amylase, to tissue damage such as lactate and lactate dehydrogenase (LDH), to sepsis such as aldolase and biomarkers of redox status such the ferric-reducing ability of saliva (FRAS), and advanced oxidation protein products (AOPP) will be evaluated. With the results of this panel, the ability of the different analytes to differentiate between the animals with disease and healthy individuals would be analyzed, and the correlation between these analytes and calgranulins will be studied.

## 2. Materials and Methods

### 2.1. Animals

In this study, crossbred (Pietrain × Large White × Landrace) weaning pigs from 4 to 8 weeks old from a commercial farm located in Spain were included. A total of 72 pigs were divided into two groups: (1) a group of pigs with naturally occurring diarrhea caused by *E. coli* (n = 34, 18 males and 16 females) and (2) a group of clinically healthy pigs that did not show any clinical sign at external examination (n = 38, 17 males and 21 females). Pigs were placed in pens containing a standard feeder and a nipple drinker to provide ad libitum access to feed and water with a minimum space of 0.65 m2 per animal (Council Directive 2001/88/CE of 23 October 2001 amending Directive 91/630/CEE concerning minimum standards for the protection of pigs) and an average temperature of 24 ± 2 °C. All animals of the diseased group showed moderate to severe clinical signs compatible with the diarrheic syndrome (diarrhea, lethargy, growth retardation, and dehydration), and rectal swabs were used to detect the presence of *E. coli* through standard analytical procedures [21], being positive for *E. coli* F4 and heat-labile toxin. The sick pigs were identified by visual inspection in the pens, finding the signs of disease indicated above. The pigs of the healthy group were negative for the *E. coli* F4 and heat-labile toxin.

### 2.2. Saliva Sampling

To collect saliva from the pigs, a metal rod with a sponge affixed to it was used. Afterward, the sponges were taken out of the pigs’ mouths and put into Salivette tubes (manufactured by Sarstedt, Aktiengesellschaft & Co., Nümbrecht, Germany). The samples were kept at a temperature between 4 and 8 °C until they arrived at the laboratory in less than three hours after their obtention in all cases. The Salivette tubes were then centrifuged at 3000× *g* and at a temperature of 4 °C for 10 min to collect the saliva supernatant. The aliquots were dispensed to Eppendorf tubes and stored at a temperature of −80 °C until the analysis was performed.

In the group of sick pigs, the saliva samples were obtained on the first day on which clinical signs of the diarrheic syndrome were observed and before the administration of any treatment.

### 2.3. Biochemical Analysis of Saliva

#### 2.3.1. Measurements of S100A8/A9 and S100A12

S100A8/A9 was analyzed with the BÜHLMANN fCal Turbo^®^ assay kit (BÜHLMANN, Laboratories AG, Schönenbuch, Switzerland) using an Olympus AU400 autoanalyzer. This assay is designed for humans, but it has been demonstrated to have cross-reactivity with calprotectin in pigs [22] and has been validated for use in the saliva of this species [14]. The description of this assay and the other automated assays used in this study appears in Supplementary Table S1.

SA100A12 was measured by a two-step direct sandwich assay developed in 96-well plates with a total sample volume of 5 μL per well using the AlphaLisa technology. The assay was performed with a commercial polyclonal antibody against porcine SA100A12 (abx129972, Abbexa Ltd., Cambridge, UK) conjugated to 1 mg of acceptor beads (AlphaScreen^®^ Unconjugated Acceptor Beads, PerkinElmer, Waltham, MA, USA) and with the same polyclonal antibody biotinylated with a 20-fold molar excess (EZ-Link™, Micro Sulfo-NHS-Biotin, No-Weight™ Format, Thermo Scientific, Waltham, MA, USA). To optimize the assay, different concentrations of biotinylated antibodies (0, 0′3, 0′6, 1, 3, and 6 nM), acceptor beads (5, 10, 20, and 30 µg/mL), and donor beads (10, 20, and 40 µg/mL) were tested. A saliva sample of known concentration measured with a specific commercially available assay (SED080Po Cloud-Clone, Katy, TX, USA) was used as a standard. This commercial assay was analytically validated for use in pig saliva samples in a previous report [17]. The AlphaLisa assay provided an intra- and inter-assay imprecision of <15% and was linear after serial sample dilution. The analytical validation data of this assay can be found in the supplementary data (Tables S2–S4 and Figure S1). Results are expressed in mg/L.

#### 2.3.2. Biomarkers of Inflammation

Haptoglobin concentrations were measured by an assay based on AlphaLisa technology previously used in pig saliva [23]. The ITIH4 was determined using a porcine species-specific commercially available ELISA kit (Porcine ITIH4, ElabSciences, Houston, TX, USA). The kit showed maximum values of intra and inter-imprecision lower than 15% and was linear after serial sample dilution for quantifying the ITIH4 in pig saliva [17].

Additionally, total protein concentration was determined by a commercial colorimetric kit to measure urine and low-complexity region (LCR) proteins (protein in urine and CSF, Spinreact, Girona, Spain) that have been previously validated in pig saliva [24], in an autoanalyzer Olympus AU400 (Beckman Olympus AU400 Chemistry Analyzer, Beckman Coulter, Brea, CA, USA). 

#### 2.3.3. Biomarker of the Immune System

ADA was analyzed with a commercially available spectrophotometric automated assay (Adenosine Deaminase assay kit, Diazyme Laboratories, Poway, CA, USA), previously validated for porcine saliva [25].

#### 2.3.4. Biomarker of Stress

The salivary alpha-amylase activity was measured by a commercial method (a-Amylase, OSR6182, Beckman Coulter) previously validated in porcine saliva [26] in an Olympus AU400 autoanalyzer (Beckman Olympus AU400 Chemistry Analyzer, Beckman Coulter, Brea, CA, USA).

#### 2.3.5. Biomarkers of Tissue Damage

LDH was measured by a commercial kit from Biosystem (Biosystem S.A., Barcelona, Spain) that was previously validated in the saliva of pigs [27]. Lactate concentrations were measured using a commercial kit from Beckman (Beckman Coulter Inc., Fullerton, CA, USA). This assay provided an intra- and inter-assay imprecision of <15% and was linear after serial sample dilution s. These methods were performed using an Olympus AU400 autoanalyzer (Beckman Olympus AU400 Chemistry Analyzer, Beckman Coulter, Brea, CA, USA).

#### 2.3.6. Biomarkers of Sepsis

Aldolase activity was determined through a commercially available reagent kit (Aldolase, Randox Laboratories Ltd., Crumlin, UK) in an Olympus AU400 autoanalyzer (Beckman Olympus AU400 Chemistry Analyzer, Beckman Coulter, Brea, CA, USA), previously validated in pigs’ saliva [15].

#### 2.3.7. Biomarkers of Redox Status

Two different biomarkers were selected to assess the redox status in the saliva of pigs: FRAS and AOPP. FRAS was determined by the reduction of ferric-tripyridyltriazine (Fe^3+^-TPTZ, Sigma-Aldrich Co., San Luis, MI, USA) to the ferrous (Fe^2+^) form [28] and the AOPP based on a method previously described [29], and both were previously used in pig saliva [30]. These analyses were carried out using an Olympus AU400 autoanalyzer (Beckman Olympus AU400 Chemistry Analyzer, Beckman Coulter, Brea, CA, USA).

### 2.4. Statistical Analysis

The distribution of each variable was assessed by the Shapiro–Wilk test showing a *p*-value < 0.05 in all parameters and by observation of QQ plots that showed that data were not close to forming a diagonal line. Therefore, the data were considered non-normal distributed and a non-parametric approach was followed to analyze all the results. For group comparison, the Mann–Whitney test was used to assess the differences in each variable. Results were expressed as median and range. Each variable’s receiver operating characteristic (ROC) curve was calculated to determine the cut-off that distinguished between groups. The curves were constructed by plotting sensitivity against 1-specificity. Those analytes showing a significant area under the curve (AUC) were selected to calculate the cut-off values by the point on the ROC curve with the minimum distance from the left-upper corner of the unit square as previously described [31]. A correlation study between the different analytes evaluated was performed through the Spearman test for non-parametric data. Results were considered significant for an alpha of 0.05. The degree of correlation was evaluated using the Rule of Thumb [32], which categorizes an R-value of 0.90 to 1 as indicative of a very high correlation, 0.70 to 0.90 a high correlation, 0.50 to 0.70 a moderate correlation, 0.30 to 0.50 a low correlation and less than 0.30 a little if any correlation.

The statistical analysis was completed by the GraphPad Prism 9 software for Mac Version 9.5.9 (GraphPad Software, LLC, San Diego, CA, USA). The statistical power of the results was calculated by a posthoc analysis using G-Power (Dusseldorf, Germany).

## 3. Results

### 3.1. Variations of S100A8/A9 and S100A12 in the Saliva of Pigs with Diarrhea Due to E. coli

**Figure 1:**
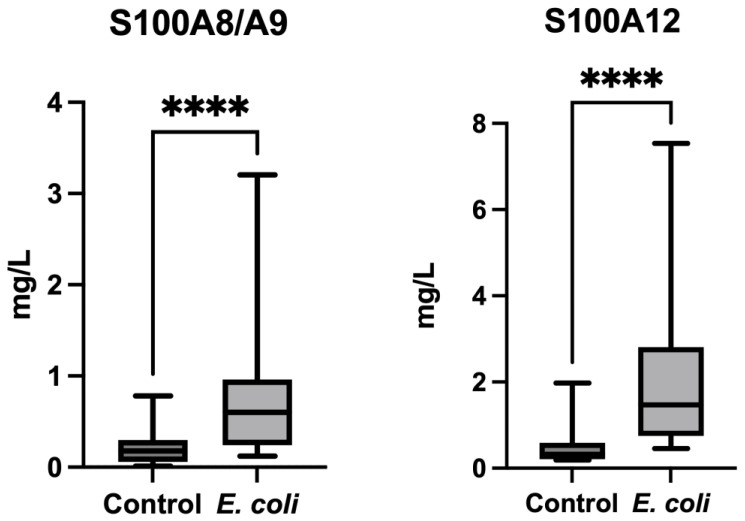
Changes in the salivary levels of S100A8/A9 (**left**) and S100A12 (**right**) in the group of pigs with diarrhea due to *E. coli* (*E. coli*) compared with healthy pigs (Control). Boxes show the interquartile range (25th and 75th percentile), the line within the boxes the median values, and the whiskers indicate the range (minimum and maximum value). **** *p* < 0.0001.

Diarrhea due to *E.coli* F4 and heat-labile toxin positive caused an increase in the two S100 proteins (S100A8/A9 and S100A12) in saliva (Figure 1). The concentrations of S100A8/A9 were significantly higher in the group of pigs with diarrhea due to *E. coli* (median = 0.60 mg/L; range = 0.12–3.2) compared with the group of healthy pigs (median = 0.18 mg/L; range = 0.01–0.78) (*p* < 0.001). In the case of S100A12, it was also increased in the saliva of pigs with diarrhea (median = 1.4 mg/L; range = 0.4–7.5) compared with healthy pigs (median = 0.3 mg/L; range = 0.1–1.9) (*p* < 0.001).

### 3.2. Changes of Other Salivary Analytes in Pigs with Diarrhea due to E. coli

The results (median and range) of other salivary biomarkers are presented in Table 1. In the group of biomarkers related to inflammation, haptoglobin, ITIH4, and total protein concentration increased in the group of pigs with diarrhea 3.08, 2.87, and 2.11-fold, respectively, compared with healthy pigs.

The immune system marker, ADA, showed increments of 5.46-fold in pigs with diarrhea compared with healthy pigs. In the case of the analyte related to stress, salivary alpha-amylase was 4.93-fold higher in the group of pigs with diarrhea. The two analytes included to evaluate the presence of tissue damage, lactate, and LDH, showed an increase of 3.83 and 2.86-fold in the group of pigs with diarrhea compared with healthy pigs. In the marker of sepsis, aldolase, an increase of 3.36-fold was observed in the saliva of pigs with diarrhea compared with healthy pigs. Finally, the two markers included in this panel to evaluate the redox status, FRAS, and AOPP, also showed a 1.85 and 2.31-fold increase in the pigs with diarrhea compared with controls.

The ROC curve analyses showed AUCs of 0.88 and 0.81 for S100A12 and S100A8/A9, respectively. Additionally, AUCs higher than 0.8 were observed for ADA (AUC = 0.87), aldolase (AUC = 0.87), total proteins (AUC = 0.87), ITIH4 (AUC = 0.85), and haptoglobin (AUC = 0.84) (Table 2).

### 3.3. Correlation of Salivary Analytes

Spearman correlation analysis showed a moderate positive correlation of S100A8/A9 with ADA (r = 0.61; *p* < 0.001) and ITIH4 (r = 0.50; *p* = 0.002). The S100A12 showed a high positive correlation with total proteins (r = 0.81; *p* < 0.001), and AOPP (r = 0.75; *p* < 0.001), and a moderate positive correlation with FRAS (r = 0.69; *p* < 0.001), LDH (0.63; *p* < 0.001), ITIH4 (r = 0.65; *p* < 0.001), ADA (r = 0.58; *p* < 0.001), and lactate (r = 0.54; *p* < 0.001). A significant moderate correlation (r = 0.65; *p* < 0.001) was found between the two S100s proteins (S100A8/A9 and S100A12).

## 4. Discussion

In this report, increases in the calgranulins S100A8/A9 and S100A12 in saliva were found in pigs with diarrhea caused by *E. coli* F4 and heat-labile toxin positive. In addition, other analytes related to inflammation, the immune system, stress, tissue damage, sepsis, and redox status showed increases in saliva in this disease. To our knowledge, this is the first report in which S100A8/A9 and S100A12, as well as a comprehensive profile of salivary analytes, are evaluated in diseased pigs with diarrhea due to *E. coli.*

An AlphaLisa assay that was developed in-house with a commercially available polyclonal antibody was used for the quantification of S100A12 in this report. The use of this assay instead of an existing commercially available ELISA (SED080Po Cloud-Clone, Katy, TX, USA) was preferred in order to gain the advantages of the AlphaLisa technology such as the use of low sample volume and the lack of need for washing steps, with the consequent saving of time as it has only two incubation steps and a total test time of 2 h. In the analytical validation study, this new assay was precise and linear and demonstrated to be suitable for use in pigs saliva. However, it should be considered that the use of other methods or assays could provide different values. This has been described in humans in the case of S100A8/A9 with different methods such as fluorometric assays, turbidimetric assays, lateral flow tests, and chemiluminescent or enzyme-linked immunoassays giving different values [33,34,35]. Based on this, it would be recommended that, in any case, each different assay should be validated from an analytical point of view in the species and sample type to be used before its application. In the case of assays for the measurement of S100A8/A9 and S100A12 used in the present study, saliva samples with a known concentration of each analyte were used as calibrators to take the advantage of reducing the matrix effect [14].

Increases of both calgranulins measured (S100A8/A9 and S100A12) were found in the saliva of pigs with diarrhea due to *E. coli* in our study. To our knowledge, there are no reports about changes in S100A8/S100A9 or S100A12 in saliva in diseases related to diarrhea or intestinal disease in pigs. However, there is evidence that both proteins increase in these situations in feces and serum in humans. For example, both proteins were increased in feces in patients with the intestinal disease [36,37]. Also, serum levels of S100A8/S100A9 showed their utility in monitoring the presence of inflammation in patients with Crohn’s disease or ulcerative colitis [38], and S100A12 concentrations in the serum of patients with inflammatory bowel disease are correlated with disease activity [39]. In humans, it is postulated that the presence in the serum of the calgranulins could be derived from their massive expression described in inflamed tissue from patients with active inflammatory bowel disease [39] and the increased number of neutrophils and other inflammatory cells that can be found in the tissue which are a rich source of them [36]. In pigs, it is interesting to point out that in an experimental challenge with ETEC, the S100A/A9 expression in jejunal mucosa increased after the challenge and was reduced after treatment and the improvement of intestinal inflammation [40]. Therefore, S100A8/A9 could have the potential for treatment monitoring. Further studies should be made to elucidate the reasons for the increases of S100A8/A9, and S100A12 found in the saliva of pigs with diarrhea due to *E. coli* in our report and clarify if they could come from serum and/or be expressed in saliva as it has been described in mice [41]. In this line, it should be indicated that the increases in these analytes could also be influenced by the loss of organic water leading to more concentrated saliva during diarrhea. Further studies should be made to elucidate and clarify the possible influence of water losses on the values of the analytes in saliva.

Regarding the other analytes evaluated in this study, the three biomarkers of inflammation measured (haptoglobin, ITIH4, and total protein concentration) showed an increase in the saliva of the diseased pigs. This could indicate an inflammation associated with diarrhea caused by *E. coli* that could be detected by these biomarkers. Increases in serum haptoglobin and ITIH4 have been described in pigs experimentally infected with *E. coli* [42,43]. Haptoglobin was 3.8-fold higher in the saliva of pigs with diarrhea in our study, which is consistent with the performance of haptoglobin as a moderate acute phase protein in swine [44,45]. In the present study, the mean increase of ITIH4 was 2.87-fold, in line with the magnitude of increases reported in the serum of pigs with *E. coli* infection compared to healthy pigs [42]. The activity of ADA showed an increase in pigs with diarrhea in our study, in line with a previous report [20] in which increments in this protein in saliva were detected by proteomics and a spectrophotometric assay in pigs with the same disease. The increase of alpha-amylase in this report could be due to the described role of an increase in salivary alpha-amylase as an evolutive adaptation in order to protect the organism against gastrointestinal pathogens [46]. The increases in lactate and LDH could reflect poor perfusion and tissue damage, Also, aldolase was found to increase in the diseased animals; this could reflect a state of sepsis and would agree with the results of a previous report where pigs were individually administered LPS from *E. coli* and showed increases in this enzyme both by proteomics and spectrophotometric assays [15]. In addition, the two biomarkers of oxidative stress that increase in this report, FRAS, and AOPP, were also increased in the saliva of pigs experimentally infected with LPS from *E. coli* [16].

The two calgranulins measured in this study presented values of AUC > 0.8 at ROC curve analysis; therefore, they could potentially distinguish between healthy pigs and pigs with *E. coli* infection. In addition, other analytes showed AUC higher than 0.8 such as ADA, aldolase, total protein, ITIH4, and haptoglobin. It should be pointed out that in most cases there was an overlap of the values of the analytes between both groups and due to the non-specific nature of these markers, they cannot be diagnostics of the disease, and specific methods for *E. coli* detection are needed for this purpose. However, these analytes could have other possible practical applications in this disease. For example, they could allow early detection of the disease and predict its appearance in the near future in animals without clinical signs and also could be useful for treatment monitoring.

In this study, S100A8/A9 and S100A12 showed a positive correlation. This correlation was lower than others previously described in human serum in patients with septic shock and inflammatory bowel disease [47,48] and in saliva of pigs with meningitis due to *S. suis* [17]; further studies should be conducted to determine if the correlation between these two proteins could vary depending on the disease. These proteins showed significant correlations with the APP ITIH4 in our study, indicating their involvement in the inflammatory process. Calgranulins participate in the inflammatory response being released by neutrophils, macrophages, and monocytes to induce inflammatory cytokines. Correlations of a similar magnitude to those found in our study have been described between serum S100A8/A9 and other APPs such as CRP (r = 0.55) in patients with rheumatoid arthritis [49]. In our report, S100A8/A9 and S100A12 also showed a correlation with ADA that could be related to the ability that the S100 proteins family has to activate immune cells [47,50]. The positive correlations that S100A12 showed with AOPP and FRAS could indicate a possible role of this protein in the redox status. In this line, it was recently described that S100A12 overexpression can promote oxidative stress [51,52].

Our study has various limitations that should be pointed out. Ideally, experimental infection with *E. coli* should have been performed to study the changes of these analytes in a more controlled environment; however, it was preferred to study the disease under farm conditions. In addition, the results of this report have been obtained with F4 *E. coli,* and it would be interesting to evaluate other different serotypes. Although the statistical power obtained in this report was higher than 0.8, indicating that from a statistical point of view, the number of animals included in this study was adequate, it would also be interesting to evaluate these analytes in larger populations and also different farms. ROC analysis to evaluate the sensitivity and specificity of different analytes alone or in combination should also be made in these studies. In addition, it would also be interesting to evaluate the potential of these analytes to distinguish pigs with diarrhea due to *E. coli* from pigs with other diseases especially those causing diarrhea and not only with healthy animals. Also, further studies should be carried out to explore the possible application of these markers for early detection and treatment monitoring of the disease.

## 5. Conclusions

It can be concluded that S100A8/A9 and S100A12 increase in pigs with diarrhea produced by *E. coli* F4 and heat-labile toxin positive. In addition, in this disease, there are increases in analytes related to inflammation such as haptoglobin, ITIH4, and total protein, to the immune system such as ADA, stress such as alpha-amylase, tissue damage such as lactate and LDH, sepsis such as aldolase and to redox status such as FRAS, and AOPP. Further studies should be made to gain knowledge about the possible practical applications of the measurements of S100 A8/A9 and S100 A12 in the saliva of pigs as a biomarker to evaluate the animals’ health and welfare.

## Figures and Tables

**Table 1:** Variations in the salivary biomarkers of pigs with diarrhea (n = 34) compared with healthy pigs (n = 36).

	Healthy Group		*E. coli* Group		
	Median	Range	Median	Range	*p* Value
**S100 proteins**					
S100A8/A9 (mg/L)	0.18	0.01–0.78	0.60	0.12–3.2	<0.001
S100A12 (mg/L)	0.3	0.02–1.9	1.4	0.4–7.5	<0.001
**Inflammation**					
Haptoglobin (mg/L)	2.08	0.36–6.85	6.41	1.69–33.84	<0.001
ITIH4 (µg/L))	8.97	1.24–34.63	25.81	3.26–111.1	<0.001
Total protein (mg/dL)	116.9	20.90–424.8	246.9	90.22–567.9	<0.001
**Immune system**					
Adenosine deaminase (U/L)	2173	32–31,328	11,600	1229–108,851	<0.001
**Stress**					
Alpha-amylase (U/L)	1221	60–52,290	5997	76.70–83,992	<0.001
**Tissue damage**					
Lactate (mmol/L)	5.56	0.1–27.20	21.04	1.12–27.29	<0.001
LDH (U/L)	166.8	2.4–808	401.9	55.80–2093	0.001
**Sepsis**					
Aldolase (U/L)	8.65	1.80–44.40	29.10	6.60–131.6	<0.001
**Redox status**					
FRAS (µmol/L)	210	70–650	370	130–1460	<0.001
AOPP (µmol/L)	128	28–658.6	301.7	111.3–1031	0.002

ITIH4: Inter-alpha trypsin inhibitor 4; LDH: lactate dehydrogenase; FRAS: ferric reduction ability of saliva; AOPP: advance oxidation protein products.

**Table 2:** Receiver operating characteristic (ROC) curve analysis for determination of cutoff value for the salivary analytes investigated in pigs with diarrhea.

	Area under the ROC Curve (AUC)	95% CI	*p* Value	Cutoff Value	Sensitivity (%)	Specificity (%)
**S100 proteins**						
S100A8/A9 (mg/L)	0.81	0.72–0.91	<0.001	0.21	77.14	68.42
S100A12 (mg/L)	0.88	0.78–0.98	<0.001	0.5	90	70
**Inflammation**						
Haptoglobin (mg/L)	0.84	0.75–0.92	<0.001	3.40	80.56	69.23
ITIH4 (µg/L)	0.85	0.72–0.98	<0.001	16.90	87.5	84.21
Total protein (mg/dL)	0.87	0.79–0.95	<0.001	155.2	85.29	71.05
**Immune system**						
Adenosine deaminase (U/L)	0.87	0.79–0.95	<0.001	6214	88.24	78.38
**Stress**						
Alpha-amylase (U/L)	0.72	0.6–0.84	<0.001	1706	72.22	73.68
**Tissue damage**						
Lactate (mmol/L)	0.77	0.66–0.89	<0.001	18.8	75	70.27
LDH (U/L)	0.75	0.62–0.88	0.001	182.6	75	60.87
**Sepsis**						
Aldolase (U/L)	0.87	0.79–0.95	<0.001	13.4	88.24	73.68
**Redox status**						
FRAS (µmol/L)	0.79	0.67–0.92	<0.001	0.24	81.82	69.23
AOPP (µmol/L)	0.75	0.61–0.89	0.002	147.3	82.61	60

ITIH4: Inter-alpha trypsin inhibitor 4; LDH: lactate dehydrogenase; FRAS: ferric reduction ability of saliva; AOPP: advance oxidation protein products.

## Data Availability

Data is available upon reasonable request.

## Supplementary Materials

The following supporting information can be downloaded at: https://www.mdpi.com/article/10.3390/ani13162556/s1. References [14,15,25,26,27,30,53,54] are cited in the supplementary materials.

## References

[1] Xia C., Braunstein Z., Toomey A.C., Zhong J., Rao X. (2018). S100 Proteins As an Important Regulator of Macrophage Inflammation. Front. Immunol..

[2] Cerón J.J., Ortín-Bustillo A., José López-Martínez M., Martínez-Subiela S., David Eckersall P., Tecles F., Tvarijonaviciute A., Muñoz-Prieto A. (2023). S-100 Proteins: Basics and Applications as Biomarkers in Animals with Special Focus on Calgranulins (S100A8, A9, and A12). Biology.

[3] Thames B.E., Barr J.W., Suchodolski J.S., Steiner J.M., Heilmann R.M. (2019). Prospective Evaluation of S100A12 and S100A8/A9 (Calprotectin) in Dogs with Sepsis or the Systemic Inflammatory Response Syndrome. J. Vet. Diagn. Investig..

[4] Chen Y., Wang C., Song J., Xu R., Ruze R., Zhao Y. (2021). S100A2 Is a Prognostic Biomarker Involved in Immune Infiltration and Predict Immunotherapy Response in Pancreatic Cancer. Front. Immunol..

[5] Meijer B., Gearry R.B., Day A.S. (2012). The Role of S100A12 as a Systemic Marker of Inflammation. Int. J. Inflamm..

[6] Gonzalez L.L., Garrie K., Turner M.D. (2020). Role of S100 Proteins in Health and Disease. Biochim. Biophys. Acta (BBA)-Mol. Cell Res..

[7] Vogl T., Pröpper C., Hartmann M., Strey A., Strupat K., van den Bos C., Sorg C., Roth J. (1999). S100A12 Is Expressed Exclusively by Granulocytes and Acts Independently from MRP8 and MRP14. J. Biol. Chem..

[8] Zackular J.P., Chazin W.J., Skaar E.P. (2015). Nutritional Immunity: S100 Proteins at the Host-Pathogen Interface. J. Biol. Chem..

[9] Zimmer D.B., Eubanks J.O., Ramakrishnan D., Criscitiello M.F. (2013). Evolution of the S100 Family of Calcium Sensor Proteins. Cell Calcium.

[10] Kim J.W., Jung J.Y., Lee S.W., Baek W.Y., Kim H.A., Suh C.H. (2022). S100A8 in Serum, Urine, and Saliva as a Potential Biomarker for Systemic Lupus Erythematosus. Front. Immunol..

[11] Russo A., Schürmann H., Brandt M., Scholz K., Matos A.L.L., Grill D., Revenstorff J., Rembrink M., von Wulffen M., Fischer-Riepe L. (2022). Alarming and Calming: Opposing Roles of S100A8/S100A9 Dimers and Tetramers on Monocytes. Adv. Sci..

[12] Cerón J.J., Contreras-Aguilar M.D., Escribano D., Martínez-Miró S., López-Martínez M.J., Ortín-Bustillo A., Franco-Martínez L., Rubio C.P., Muñoz-Prieto A., Tvarijonaviciute A. (2022). Basics for the Potential Use of Saliva to Evaluate Stress, Inflammation, Immune System, and Redox Homeostasis in Pigs. BMC Vet. Res..

[13] López-Martínez M.J., Beletić A., Kuleš J., Rešetar-Maslov D., Rubić I., Mrljak V., Manzanilla E.G., Goyena E., Martínez-Subiela S., Cerón J.J. (2022). Revealing the Changes in Saliva and Serum Proteins of Pigs with Meningitis Caused by Streptococcus Suis: A Proteomic Approach. Int. J. Mol. Sci..

[14] López-Martínez M.J., Martínez-Subiela S., Cerón J.J., Ortín-Bustillo A., Ramis G., López-Arjona M., Martínez-Miró S., Manzanilla E.G., Eckersall P.D., Tecles F. (2023). Measurement of Calprotectin (S100A8/A9) in the Saliva of Pigs: Validation Data of A Commercially Available Automated Assay and Changes in Sepsis, Inflammation, and Stress. Animals.

[15] López-Martínez M.J., Cerón J.J., Ortín-Bustillo A., Escribano D., Kuleš J., Beletić A., Rubić I., González-Sánchez J.C., Mrljak V., Martínez-Subiela S. (2022). A Proteomic Approach to Elucidate the Changes in Saliva and Serum Proteins of Pigs with Septic and Non-Septic Inflammation. Int. J. Mol. Sci..

[16] López-Martínez M.J., Escribano D., Ortín-Bustillo A., Franco-Martínez L., González-Arostegui L.G., Cerón J.J., Rubio C.P. (2022). Changes in Biomarkers of Redox Status in Saliva of Pigs after an Experimental Sepsis Induction. Antioxidants.

[17] López-Martínez M.J., Ornelas M.A.S., Amarie R.E., Manzanilla E.G., Martínez-Subiela S., Tecles F., Tvarijonaviciute A., Escribano D., González-Bulnes A., Cerón J.J. (2023). Changes in Salivary Biomarkers of Stress, Inflammation, Redox Status, and Muscle Damage Due to Streptococcus Suis Infection in Pigs. BMC Vet. Res..

[18] Majster M., Almer S., Boström E.A. (2019). Salivary Calprotectin Is Elevated in Patients with Active Inflammatory Bowel Disease. Arch. Oral Biol..

[19] Zhu C., Lv Y., Yang J., Bai Y., Ye J., Wang Z., Chen Z., Jiang Z. (2020). Proteomic Alteration of Porcine Intestinal Epithelial Cells after Pretreatment with Lactobacillus Plantarum Followed by Infection with Enterotoxigenic Escherichia Coli F4. Vet. Immunol. Immunopathol..

[20] Rodrigues M., López-Martinez M.J., Ortin-Bustillo A., Cerón J.J., Martinez-Subiela S., Muñoz-Prieto A., Lamy E. (2023). Changes in the Saliva Proteome of Pigs with Diarrhoea Caused by Escherichia Coli. Proteomes.

[21] Mesonero-Escuredo S., Strutzberg-Minder K., Casanovas C., Segalés J. (2018). Viral and Bacterial Investigations on the Aetiology of Recurrent Pig Neonatal Diarrhoea Cases in Spain. Porc. Health Manag..

[22] Barbosa J.A., Rodrigues L.A., Columbus D.A., Aguirre J.C.P., Harding J.C.S., Cantarelli V.S., Costa M. (2021). de O. Experimental Infectious Challenge in Pigs Leads to Elevated Fecal Calprotectin Levels Following Colitis, but Not Enteritis. Porc. Health Manag..

[23] Contreras-Aguilar M.D., López-Arjona M., Martínez-Miró S., Escribano D., Hernández-Ruipérez F., Cerón J.J., Tecles F. (2021). Changes in Saliva Analytes during Pregnancy, Farrowing and Lactation in Sows: A Sialochemistry Approach. Vet. J..

[24] Tecles F., Escribano D., Martinez-Miro S., Ceron J.J. (2017). Homocysteine Measurement in Pig Saliva, Assay Validation and Changes after Acute Stress and Experimental Inflammation Models: A Pilot Study. Res. Vet. Sci..

[25] Tecles F., Rubio C.P., Contreras-Aguilar M.D., Lopez-Arjona M., Martinez-Miro S., Martinez-Subiela S., Ceron J.J. (2018). Adenosine Deaminase Activity in Pig Saliva: Analytical Validation of Two Spectrophotometric Assays. J. Vet. Diagn. Investig..

[26] Fuentes M., Tecles F., Gutiérrez A., Otal J., Martínez-Subiela S., Cerón J.J. (2011). Validation of an Automated Method for Salivary Alpha-Amylase Measurements in Pigs (Sus Scrofa Domesticus) and Its Application as a Stress Biomarker. J. Vet. Diagn. Investig..

[27] Escribano D., Horvatić A., Contreras-Aguilar M.D., Guillemin N., Cerón J.J., Tecles F., Martinez-Miró S., Eckersall P.D., Manteca X., Mrljak V. (2019). Changes in Saliva Proteins in Two Conditions of Compromised Welfare in Pigs: An Experimental Induced Stress by Nose Snaring and Lameness. Res. Vet. Sci..

[28] Benzie I.F.F., Strain J.J. (1996). The Ferric Reducing Ability of Plasma (FRAP) as a Measure of “Antioxidant Power”: The FRAP Assay. Anal. Biochem..

[29] Witko-Sarsat V., Friedlander M., Capeillère-Blandin C., Nguyen-Khoa T., Nguyen A.T., Zingraff J., Jungers P., Descamps-Latscha B. (1996). Advanced Oxidation Protein Products as a Novel Marker of Oxidative Stress in Uremia. Kidney Int..

[30] Rubio C.P., Mainau E., Cerón J.J., Contreras-Aguilar M.D., Martínez-Subiela S., Navarro E., Tecles F., Manteca X., Escribano D. (2019). Biomarkers of Oxidative Stress in Saliva in Pigs: Analytical Validation and Changes in Lactation. BMC Vet. Res..

[31] Perkins N.J., Schisterman E.F. (2006). The Inconsistency of “Optimal” Cutpoints Obtained Using Two Criteria Based on the Receiver Operating Characteristic Curve. Am. J. Epidemiol..

[32] Hinkle D., Wiersma W., Jurs S.G. (2003). Applied Statistics for Behavioral Sciences.

[33] Oyaert M., Boel A., Jacobs J., Bremt S.V.D., De Sloovere M., Vanpoucke H., Van Hoovels L. (2017). Van Analytical Performance and Diagnostic Accuracy of Six Different Faecal Calprotectin Assays in Inflammatory Bowel Disease. Clin. Chem. Lab. Med. (CCLM).

[34] Juricic G., Brencic T., Kuna A.T., Njegovan M., Honovic L. (2019). Faecal Calprotectin Determination: Impact of Preanalytical Sample Treatment and Stool Consistency on within- and between-Method Variability. Biochem. Med..

[35] Labaere D., Smismans A., Van Olmen A., Christiaens P., D’haens G., Moons V., Cuyle P.-J., Frans J., Bossuyt P. (2014). Comparison of Six Different Calprotectin Assays for the Assessment of Inflammatory Bowel Disease. United Eur. Gastroenterol. J..

[36] Pruenster M., Vogl T., Roth J., Sperandio M. (2016). S100A8/A9: From Basic Science to Clinical Application. Pharmacol. Ther..

[37] Ikhtaire S., Shajib M.S., Reinisch W., Khan W.I. (2016). Fecal Calprotectin: Its Scope and Utility in the Management of Inflammatory Bowel Disease. J. Gastroenterol..

[38] Boehm D., Krzystek-Korpacka M., Neubauer K. (2009). Paraoxonase-1 Status in Crohn’s Disease and Ulcerative Colitis. Inflamm. Bowel Dis..

[39] Foell D. (2003). Expression of the Pro-Inflammatory Protein S100A12 (EN-RAGE) in Rheumatoid and Psoriatic Arthritis. Rheumatology.

[40] Xiao D., Wang Y., Liu G., He J., Qiu W., Hu X., Feng Z., Ran M., Nyachoti C.M., Kim S.W. (2014). Effects of Chitosan on Intestinal Inflammation in Weaned Pigs Challenged by Enterotoxigenic Escherichia Coli. PLoS ONE.

[41] Javkhlan P., Hiroshima Y., Azlina A., Hasegawa T., Yao C., Akamatsu T., Kido J., Nagata T., Hosoi K. (2009). Induction of Calprotectin MRNAs by Lipopolysaccharide in the Salivary Gland of Mice. J. Med. Investig..

[42] Wong B.T., Park S., Kovanda L., He Y., Kim K., Xu S., Lingga C., Hejna M., Wall E., Sripathy R. (2022). Dietary Supplementation of Botanical Blends Enhanced Performance and Disease Resistance of Weaned Pigs Experimentally Infected with Enterotoxigenic *Escherichia Coli* F18. J. Anim. Sci..

[43] López-Colom P., Yu K., Barba-Vidal E., Saco Y., Martín-Orúe S.M., Castillejos L., Solà-Oriol D., Bassols A. (2019). I-FABP, Pig-MAP and TNF-α as Biomarkers for Monitoring Gut-Wall Integrity in Front of Salmonella Typhimurium and ETEC K88 Infection in a Weaned Piglet Model. Res. Vet. Sci..

[44] López-Martínez M.J., Franco-Martínez L., Martínez-Subiela S., Cerón J.J. (2022). Biomarkers of Sepsis in Pigs, Horses and Cattle: From Acute Phase Proteins to Procalcitonin. Anim. Health Res. Rev..

[45] Cerón J.J., Tecles F., Escribano D., Fuentes-Pardo P., Martínez-Subiela S. (2016). Acute Phase Proteins in Pigs: From Theory to Practice. Suis.

[46] Pruimboom L., Fox T., Muskiet F.A.J. (2014). Lactase Persistence and Augmented Salivary Alpha-Amylase Gene Copy Numbers Might Have Been Selected by the Combined Toxic Effects of Gluten and (Food Born) Pathogens. Med. Hypotheses.

[47] Dubois C., Marcé D., Faivre V., Lukaszewicz A.-C., Junot C., Fenaille F., Simon S., Becher F., Morel N., Payen D. (2019). High Plasma Level of S100A8/S100A9 and S100A12 at Admission Indicates a Higher Risk of Death in Septic Shock Patients. Sci. Rep..

[48] Leach S.T., Yang Z., Messina I., Song C., Geczy C.L., Cunningham A.M., Day A.S. (2007). Serum and Mucosal S100 Proteins, Calprotectin (S100A8/S100A9) and S100A12, Are Elevated at Diagnosis in Children with Inflammatory Bowel Disease. Scand. J. Gastroenterol..

[49] Hurnakova J., Hulejova H., Zavada J., Hanova P., Komarc M., Mann H., Klein M., Sleglova O., Olejarova M., Forejtova S. (2017). Relationship between Serum Calprotectin (S100A8/9) and Clinical, Laboratory and Ultrasound Parameters of Disease Activity in Rheumatoid Arthritis: A Large Cohort Study. PLoS ONE.

[50] Cesaro A., Anceriz N., Plante A., Pagé N., Tardif M.R., Tessier P.A. (2012). An Inflammation Loop Orchestrated by S100A9 and Calprotectin Is Critical for Development of Arthritis. PLoS ONE.

[51] Zhang X., Shen R., Shu Z., Zhang Q., Chen Z. (2020). S100A12 Promotes Inflammation and Apoptosis in Ischemia/Reperfusion Injury via ERK Signaling In Vitro Study Using PC12 Cells. Pathol. Int..

[52] Xie J., Luo C., Mo B., Lin Y., Liu G., Wang X., Li L. (2022). Inflammation and Oxidative Stress Role of S100A12 as a Potential Diagnostic and Therapeutic Biomarker in Acute Myocardial Infarction. Oxid. Med. Cell Longev..

[53] Orsonneau J.L., Douet P., Massoubre C., Lustenberger P., Bernard S. (1989). An improved pyrogallol red-molybdate method for determining total urinary protein. Clin. Chem..

[54] Hutchesson A., Preece M.A., Gray G., Green A. (1997). Measurement of lactate in cerebrospinal fluid in investigation of inherited metabolic disease. Clin. Chem..

