# Supplementary material for: Changes in S100A8/A9 and S100A12 and Their Comparison with Other Analytes in the Saliva of Pigs with Diarrhea Due to E. coli
Source: Animals (Basel). 2023 Aug 8;13(16):2556. doi: 10.3390/ani13162556 (PMC10451909; doi:10.3390/ani13162556)

Table S1: Description of the automated assays performed in the study.

Analyte*	Type of assay	Substrate or antibody	Company	Reagents volume μL)			Wavelength (nm)	Ref
				Sample	R1	R2		
**S100A8/A9**	Immunoturbidimetry	Avian polyclonal antibody against human S100A8/A9	Buhlman	10	130	26	540	[1]
**Total proteins**	Spectrophotometry	Pyrogallol red and molybdate	Spinreact	2	190		580	[2]
**Adenosine deaminase**	Spectrophotometric	Adenosine	Diazyme	5	180	90	550	[3]
**Alpha-amylase**	Spectophotometry	Ethylidene-G7PNP	Beckman Coulter	2	100	25	410	[4]
**Lactate dehydrogenase**	Spectophotometry	NADH	Diazyme	6	240	60	340	[5]
**Lactate**	Spectophotometry	L-Lactate	Beckman Coulter	10	200		540	[6]
**Aldolase**	Spectrophotometry	NS**	Randox	8	150	30	340	[7]
**AOPP**	Spectophotometry	Oxidatively-modified albumin	Home-made	10	200	25	340	[8]
**FRAS**	Spectophotometry	Fe^{3+}	Home-made	10	300		600	[8]

*All assays were conducted in the Olympus AU400, at a constant temperature of 37°C; **Substrate not specified; NADH: Nicotinamide adenine dinucleotide; AOPP: advanced oxidative protein products; FRAS: ferric reduction ability of saliva.

1. López-Martínez MJ, Martínez-Subiela S, Cerón JJ, Ortín-Bustillo A, Ramis G, López-Arjona M, et al. Measurement of Calprotectin (S100A8/A9) in the Saliva of Pigs: Validation Data of A Commercially Available Automated Assay and Changes in Sepsis, Inflammation, and Stress. Animals. 2023;13:1190.

2. Orsonneau JL, Douet P, Massoubre C, Lustenberger P, Bernard S. An improved pyrogallol red-molybdate method for determining total urinary protein. Clin Chem. 1989;35:2233–6.

3. Tecles F, Rubio CP, Contreras-Aguilar MD, Lopez-Arjona M, Martinez-Miro S, Martinez-Subiela S, et al. Adenosine deaminase activity in pig saliva: analytical validation of two spectrophotometric assays. J Vet Diagn Invest. 2018;30:175–9.

4. Fuentes M, Tecles F, Gutiérrez A, Otal J, Martínez-Subiela S, Cerón JJ. Validation of an Automated Method for Salivary Alpha-Amylase Measurements in Pigs (Sus Scrofa Domesticus) and its Application as a Stress Biomarker. Journal of Veterinary Diagnostic Investigation. 2011;23:282–7.

5. Escribano D, Horvatić A, Contreras-Aguilar MD, Guillemin N, Cerón JJ, Tecles F, et al. Changes in saliva proteins in two conditions of compromised welfare in pigs: An experimental induced stress by nose snaring and lameness. Res Vet Sci. 2019;125:227–34.

6. Hutchesson A, Preece MA, Gray G, Green A. Measurement of lactate in cerebrospinal fluid in investigation of inherited metabolic disease. Clin Chem. 1997;43:158–61.

7. López-Martínez MJ, Cerón JJ, Ortín-Bustillo A, Escribano D, Kuleš J, Beletić A, et al. A Proteomic Approach to Elucidate the Changes in Saliva and Serum Proteins of Pigs with Septic and Non-Septic Inflammation. Int J Mol Sci. 2022;23:6738.

8. Rubio CP, Mainau E, Cerón JJ, Contreras-Aguilar MD, Martínez-Subiela S, Navarro E, et al. Biomarkers of oxidative stress in saliva in pigs: analytical validation and changes in lactation. BMC Vet Res. 2019;15:144.

Table S2. Intra- and inter-assay imprecision of the S100A12 assay.

	Sample concentration	Mean (mg/L)	Standard deviation (mg/L)	Coefficient of variation* (%)
Intra-assay	High	4.28	0.2	4.71
	Medium	0.87	0.02	2.33
	Low	0.19	0.01	7.54
Inter-assay	High	4.14	0.16	3.86
	Medium	0.89	0.03	3.08
	Low	0.2	0.01	4.92

*Coefficient of variation was calculated as the standard deviation divided by the mean.

Table S3. Recovery study of the S100A12 assay where two saliva samples (A and B) with know concentrations were used to reach three different S100A12 concentrations.

% Sample A	% Sample B	Expected (mg/L)	Observed (mg/L)	Recovery (%)
100	0	1.86	1.86	
75	25	1.43	1.35	97.39
50	50	0.99	0.92	95.11
25	75	0.56	0.53	95.91
0	100			

Table S4. Lower limit of quantification (LLOQ) and limit of detection (LD) for the S100A12 assay.

**Limit**	**Value (mg/L)**
LLOQ*	0.0003
LD	0.000002

*The lower limit of quantification was calculated as the lowest value that the method was able to measure with a maximum imprecision of 20%; **The limit of detection is the lowest concentration of S100A12 that the assay can distinguish from a specimen of zero value (ultrapure water), and it was calculated as a mean value plus 3 standard deviations of 12 replicate determinations.

Figure S1. Linearity under the dilution of two saliva samples (A and B) with know concentrations.

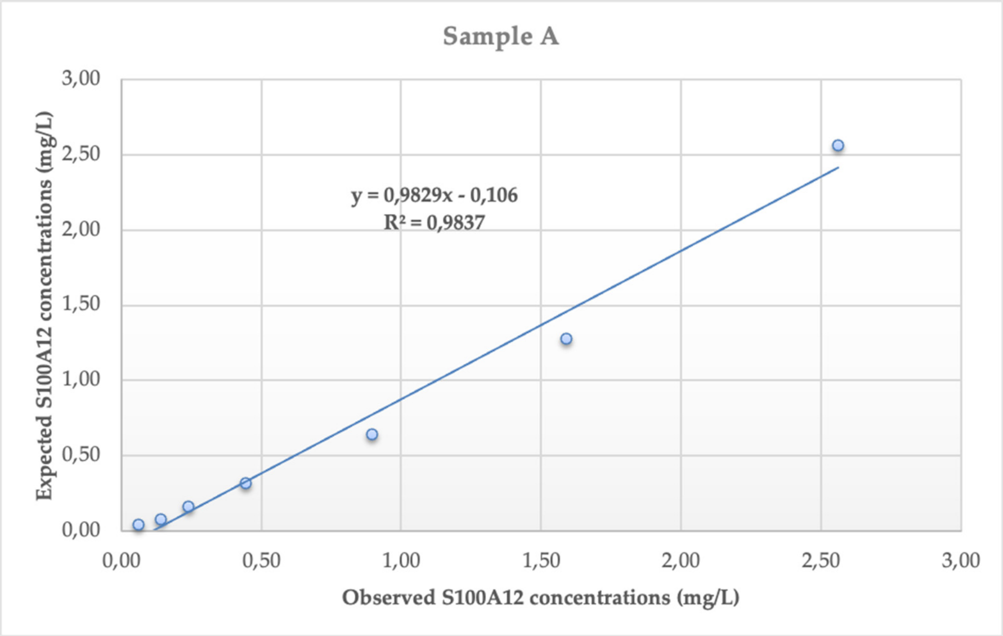

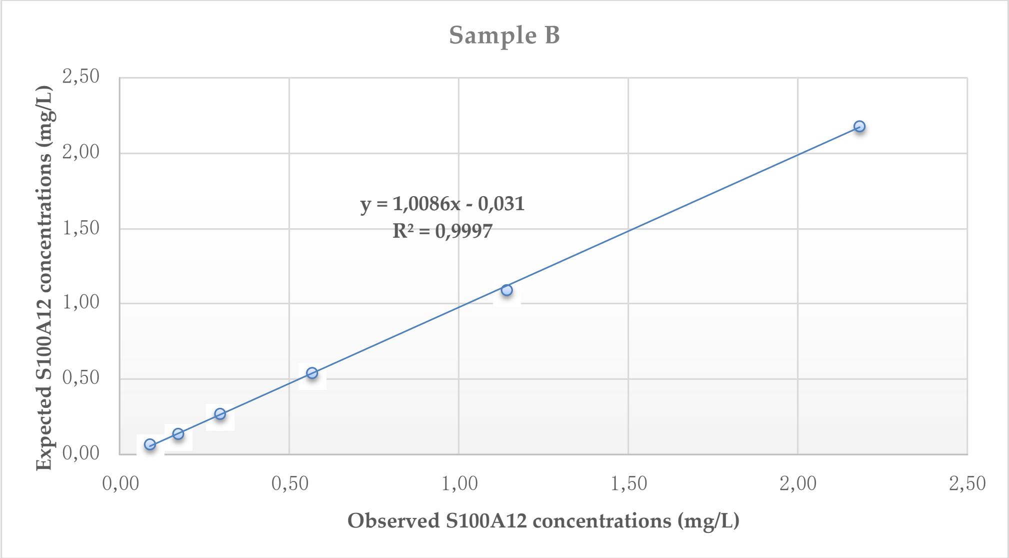

Supplement: Supplementary file 1 [file animals-13-02556-s001.zip › animals-2433037-supplementary.pdf]
